# DNA metabarcoding uncovers fungal diversity of mixed airborne samples in Italy

**DOI:** 10.1371/journal.pone.0194489

**Published:** 2018-03-20

**Authors:** Elisa Banchi, Claudio Gennaro Ametrano, David Stanković, Pierluigi Verardo, Olga Moretti, Francesca Gabrielli, Stefania Lazzarin, Maria Francesca Borney, Francesca Tassan, Mauro Tretiach, Alberto Pallavicini, Lucia Muggia

**Affiliations:** 1 Department of Life Sciences, University of Trieste, Trieste, Italy; 2 Marine Biology Station, National Institute of Biology, Piran, Slovenia; 3 Regional Agency for Environmental Protection Friuli Venezia Giulia, Department of Pordenone, Pordenone, Italy; 4 Regional Agency for Environmental Protection Umbria, Terni, Italy; 5 Regional Agency for Environmental Protection Marche, Ascoli Piceno, Italy; 6 Regional Agency for Environmental Protection Veneto, Vicenza, Italy; 7 Regional Agency for Environmental Protection Valle d’Aosta, Saint-Christophe, Italy; 8 Regional Agency for Environmental Protection Friuli Venezia Giulia, Department of Trieste, Trieste, Italy; University of Hyogo, JAPAN

## Abstract

Fungal spores and mycelium fragments are particles which become and remain airborne and have been subjects of aerobiological studies. The presence and the abundance of taxa in aerobiological samples can be very variable and impaired by changeable climatic conditions. Because many fungi produce mycotoxins and both their mycelium fragments and spores are potential allergens, monitoring the presence of these taxa is of key importance. So far data on exposure and sensitization to fungal allergens are mainly based on the assessment of few, easily identifiable taxa and focused only on certain environments. The microscopic method used to analyze aerobiological samples and the inconspicuous fungal characters do not allow a in depth taxonomical identification. Here, we present a first assessment of fungal diversity from airborne samples using a DNA metabarcoding analysis. The nuclear ITS2 region was selected as barcode to catch fungal diversity in mixed airborne samples gathered during two weeks in four sites of North-Eastern and Central Italy. We assessed the taxonomic composition and diversity within and among the sampled sites and compared the molecular data with those obtained by traditional microscopy. The molecular analyses provide a tenfold more comprehensive determination of the taxa than the traditional morphological inspections. Our results prove that the metabarcoding analysis is a promising approach to increases quality and sensitivity of the aerobiological monitoring. The laboratory and bioinformatic workflow implemented here is now suitable for routine, high-throughput, regional analyses of airborne fungi.

## Introduction

Fungi are ubiquitous and are among the most ecologically important and widespread groups of organisms which play key roles in multiple environments [1]. Fungal spores and mycelium fragments are usually so small that they belong to the group of particles that becomes and can be aerosolized (average size of 10 μm); therefore they have been investigated by aerobiologists since the early years of this field [2]. Aerobiology has been acknowledged in the 1930s as the study of biological particles in the air, including the diversity and the processes involved in the movement of microorganisms in the atmosphere between different geographical locations [3]. The long-distance dispersal of fungal spores is especially relevant for many crop plants pathogens, such as the obligatory and biotrophic fungi producing huge numbers of spores and causing, e.g. rust, blight, powdery and downy mildew diseases. Wind dispersal over hundreds or thousands of kilometers has caused the spread of these severe crop diseases on continental and even global scales [4, 5].

Besides being parasites of plants, fungi with their multiple life styles are also of general interest as they are some of the most common, severe human and clinical pathogens (e.g. [6–12]). They act as agents for a multiplicity of diseases, such as infections, toxicosis, allergic asthma, allergic rhinitis, allergic sinusitis, broncho-pulmonary mycoses, and hypersensitivity pneumonitis [6, 13–15]. Allergenic properties of spores, tissue fragments and metabolites released by fungi have been studied plentifully [6, 13, 16–20]. Because fungal aerosol in indoor environments depends in most cases from outdoor concentrations [21], the presence and distribution of allergenic fungi represents an important issue for public health [21, 22].

Researches on aerobiological samples have been performed in indoor and outdoor environments and have focused both on airborne pollen grains and fungal spores [21, 23–27]. Studies on pollen grains have developed into established monitoring networks over several countries worldwide [i.e. Italy (http://www.pollnet.it), United Kingdom (http://www.worc.ac.uk/discover/national-pollen-and-aerobiology-research-unit.html), USA (http://www.aaaai.org/global/nab-pollen-counts)]. In particular, the development of DNA barcodes for plants enhanced aerobiological analyses of plant diversity based on pollen grains [28–31]. In the past few years studies on pollen diversity have seen a large application of high throughput sequencing (HTS) technologies in palynology, melissopalynology and nutritional biology researches [32–38]. Recently Kraaijeveld et al. [38] accurately identified pollen from mixed airborne samples, including species that could not be recognized microscopically, by sequencing them with the Ion Torrent HTS platform.

On the contrary, the knowledge about fungal diversity in airborne samples is still very poor in comparison to plant data. Limitations to determine airborne fungal diversity are in part due to the very variable daily and seasonal loads of fungal (dia)spores, and to the limitations of the microscopic method used in aerobiological analysis [14, 39]. Still, aerobiology mostly employs morphological analyses of volumetric samples (usually spores/m^3^) collected with spore trapping and differentiated in non-viable and viable air sampling [21, 39, 40]. The collection of volumetric samples keeps costs low and allows the quantifications of the results. However, it usually provides only a shallow taxonomical identification of the few, most abundant and recognizable taxa, as morphological analyses suffer from being highly dependent on human expertise (i.e., it needs highly trained personnel). Indeed, routine assessments of fungal spores in pollen bulletins usually report only on a few genera, such as *Alternaria* and *Cladosporium* (https://www.pollenwarndienst.at/en/current-data/current-charts.html; http://www.isac.cnr.it/aerobio/ai/6bulletins.htm; e.g. http://www.arpa.umbria.it/pagine/spore).

In the last decade, molecular approaches (e.g., DNA barcoding, RFLP) have also been implemented to assess fungal diversity in airborne samples and have strengthen the perception that the majority of the genera were mostly overlooked by morphological inspections of either viable or non-viable samples [14]. Among the few existing studies, Pashley et al. [14] used PCR amplifications of the ITS and LSU regions coupled with cloning and sequencing of RFLP-types to show that more than two third of all genera sequenced were not detected by morphology, and that the rates were highly variable on daily basis. It was also highlighted that meteorological data, time of year, and length of the sampling period should be taken into account when comparing studies of fungal taxa with seasonality [38, 41].

Despite the wide popularity of HTS approaches in monitoring and uncovering microbial fungal diversity from diverse environments [42–47] so far very few researches applied HTS for fungal aerobiological studies. Recently, a DNA sequencing analysis was successfully implemented to identify airborne microorganisms in a hospital to control and supervise hospital infections [26]. Few other studies have assessed the composition of outdoor, aerial microbial communities (including fungi) with Illumina MiSeq [48–50] or with Roche 454 [18], showing potential for the monitoring of air pollution and human health.

**Fig 1:**
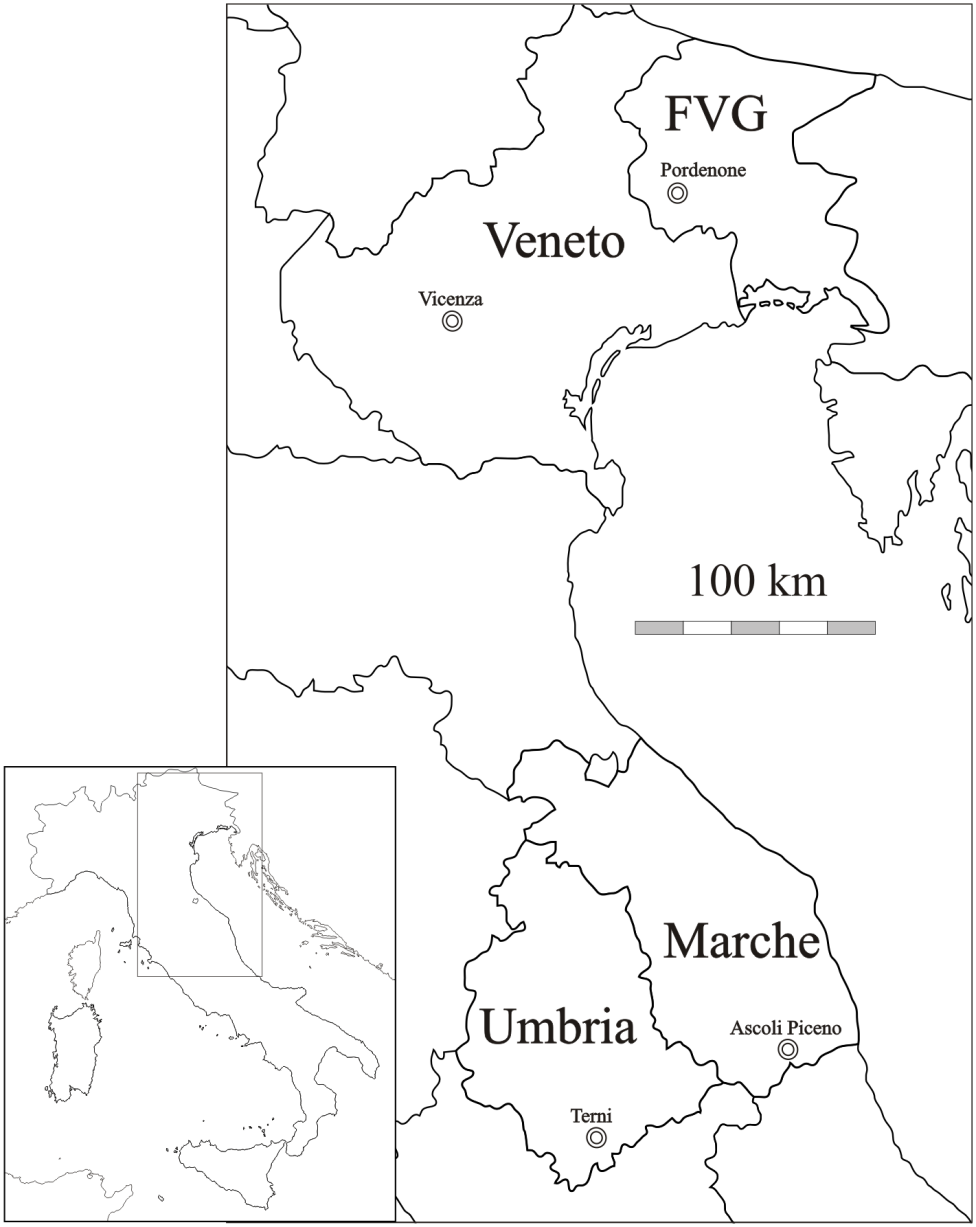
Geographical location of the sampling sites in the North-Eastern and Central Italy. Region and city names are reported (FVG: Friuli Venezia Giulia). The map has been retrieved and modified from http://www.d-maps.com.

In this study, we assessed the fungal diversity from airborne samples by implementing a DNA metabarcoding analysis using the Ion Torrent technology. We targeted the nuclear internal transcribed spacer region ITS2 and used it as fungal barcode [51–53] in mixed airborne samples collected from four sites of North-Eastern (NE) and Central Italy (Fig 1). With this approach we aimed at *i)* assessing the taxonomic composition and diversity within and among the sampled sites; *ii)* comparing the molecular data with the ones obtained by microscopy determination; *iii)* implementing a laboratory and a bioinformatic workflow suitable for routine, high-throughput, regional analyses of airborne fungi.

## Materials and methods

### Sampling

The sampling was carried out in four sites of the Italian peninsula (Fig 1) in the following regions (city): Friuli Venezia Giulia (FVG, Pordenone, 45°57'09.2"N 12°40'54.2"E, 4 m a.s.l.), Marche (Ascoli Piceno, 42°52'50.0"N 13°42'27.4"E, 154 m a.s.l.), Umbria (Terni, 42°34'48.0"N 12°37'59.4"E, 130 m a.s.l.) and Veneto (Vicenza, 45°31'49.6"N 11°35'24.2"E, 39 m a.s.l.). Sites in FVG and Veneto are in the North-Eastern Italy, those in Marche and Umbria are in Central Italy; North-Eastern sites are located about 500 km far away from the those in Central Italy. In general FVG and Veneto have lower average annual temperature (11.8°C and 11.6 °C, respectively) than Marche and Umbria (13.6°C and 12.9°C, respectively). The average annual precipitation is rather homogeneous among the sites, being 1.065 mm in FVG, 797 mm in Marche, 808 mm in Umbria and 845 mm in Veneto [54]. All sites are classified as humid-subtropical (Cfa) by the Köppen climate classification, characterized by warm temperate climate, fully humid [55].

In each site, the sampling was performed on the roof of a building at 15–20 m from the ground using a volumetric sampler (VPPS 2010, Lanzoni) mounted with a sticky tape (Melinex^®^). The sampling was performed during two whole weeks, corresponding to 5^th^-12^th^ and 19^th^-26^th^ September 2016. The sampling did not required any permission as it was performed by the cooperators of the Italian Regional Agency for Environmental Protection (ARPA, Agenzia Regionale per la Protezione all' Ambiente) for each region.

### Meteorological and land use data

Meteorological data were collected from the regional stations of ARPA FVG (located 100 m away from the sampling point of the aerobiological samples), Regione Marche (6 km), Regione Umbria (100 m), and ARPA Veneto (10 km).

Land cover data were acquired form Corine Land Cover 2012 (CLC12) data and imported in Quantum Geographic Information System (QGIS) program. A buffer of 50 km from each sampling site was used, after taking into account both the distances among sampling sites and the Mediterranean Sea. The area of the different land use was calculated on the intersection between CLC12 and the buffer.

### Microscopy analysis

The sticky tape was detached from the sampling drum under a sterile hood and cut in eight segments. Two segments at the extremities belonged to two incomplete Mondays of the weeks (the day at which the tape was weekly changed). These pieces were excluded from the molecular analyses but were microscopically inspected as representatives of the possible diversity that could have been determined by microscopy analyses alone. The eight fragments obtained in total were used to screen the presence of fungal spores and perform taxonomical identifications, which were later compared with the metabarcoding results. The sampling tapes were placed on a glass slide, mounted in water and observed at a light microscope Olympus BH-2. Fungal spore identification was based on the illustration manual of air samples of Smith [56].

### DNA extraction

Each of the remaining six segments of the tape corresponded to one full day of the week (24 h) starting from Tuesday to Sunday. Each segment was further cut into two half-day parts to fit them individually into a 1.5 ml tube, taking care that the sticky surface, on which the air samples were attached, was facing the internal part of the tube. The total DNA was extracted using the ZR Fungal/Bacterial DNA MicroPrep^™^ Kit (Zymo Research); the tapes were grinded with beads for five minutes using a bead-beater. The half-day sections were processed individually and pooled at the last step of the DNA extraction protocol to obtain a single DNA extraction for each day. This resulted in a total of 48 samples, 12 for each site.

### Molecular analysis and sequencing

The fungal nuclear ribosomal ITS2 region was amplified with the forward primer ITS3 and the reverse primer ITS4 [57]. The amplicons were obtained in two PCR amplifications. The first PCR used the ITS2 forward and reverse primers modified with GC rich universal tails on the 5’-end [58], which was identical to the tail applied on the 3’-end of the barcodes used in the second PCR. The first PCR reaction mix contained 3 μl DNA template (10–20 ng), 3 μl HotMasterMix (5PRIME), 0.5 μl BSA 10X (Sigma-Aldrich), 0.75 μl EvaGreen^™^ 20X (Biotium), 0.5 μl forward primer ITS3 (10 μM), 0.5 μl reverse primer ITS4 (10 μM) in a final volume of 15 μl. The PCR amplification was performed with CFX 96^™^ PCR System (Bio-Rad) with the following cycling profile: 94 °C for 2 min and 35 cycles at 94 °C for 20 sec, 55 °C for 20 sec, 65 °C for 40 sec followed by a final extension at 65 °C for 2 min. A negative control was used to verify the absence of non-specific amplification products and was carried out for the whole sequencing process. The second PCR (switch PCR) was required for multiplex sequencing through attachment of the barcodes. This amplification used primers modified with an 'A' adaptor and a sample-specific 10 bp barcode to the 5’-end of the forward primer, and a P1 adaptor to the 5’-end of the reverse primer. The reaction was performed in a mix containing 5 μl of the first PCR product, 20 μl HotMasterMix (5PRIME Fisher Scientific), 2.5 μl EvaGreen^™^ 20X (Biotium), 1.5 μl forward primer (10 μM), and 1.5 μl reverse primer (10 μM) in a final volume of 50 μl. PCR conditions were the same as for the first PCR but were run for 12 cycles. All the amplicons were checked for their quality and length by agarose gel electrophoresis and pooled in equimolar amount. The resulting barcoded library was run on a E-Gel Precast Agarose Electrophoresis System (Thermo Fisher Scientific). The 400 bp product was recovered, measured with Qubit^™^ Fluorimeter (Thermo Fisher Scientific) and sequenced with an Ion Torrent Personal Genome Machine (PGM, Thermo Fisher Scientific) provided with a 400 bp reads length 314^™^ chip (Thermo Fisher Scientific).

### Sequence data analysis

Data analysis was performed in QIIME 1.9.1 [59]. High quality sequences were demultiplexed, reverse primers and barcodes were removed, and reads that did not pass through the filtering (minimum length 150 bp, minimum average quality score 20, maximum length of homopolymer 8, maximum number of primer mismatches 3) were discarded. The ITS2 region was extracted with ITSx v1.0.11 [60] by selecting the fungal (F) profile option. Chimeric reads were identified and filtered out with UCHIME v4.0 algorithm using the reference dataset updated on 01.12.2016 [53, 61] to obtain the final, high quality dataset. Operational Taxonomic Units (OTUs) were picked at 97% similarity with open reference strategy and UNITE database, updated on November 2016 [62]. The method used for the taxonomic assignment was blast (max E-value 1e^-30^). Singletons were removed from the dataset. Statistics and ecological indices were performed with QIIME [59].

The alpha and beta diversity analyses were conducted on the rarefied dataset. Rarefaction threshold was set at 1284 reads, which corresponds to the number of reads in the first sample over 1000 reads. Alpha diversity in terms of OTUs richness and diversity was calculated using Chao1 [63] and Shannon indices [64]. A one-way analysis of variance (ANOVA) followed by a Duncan’s new multiple range test was applied to verify the significance of differences in alpha diversity among the sites with R version 3.2.0 [65]. The beta diversity was calculated with Bray-Curtis distances principal coordinate analysis (PCoA). As suggested in QIIME tutorial for de novo OTU picking, stability and robustness of PCoA was assessed using a resampling procedure known as jackknifing [66, 67]. For this procedure, calculations are reiterated after omitting one observation and representing the average in a PCoA plot [67]. Here, jackknife resampling was performed on the OTU table, repeating the PCoA analysis for each resampled table (with one OTU omitted) and plotting the results (i.e. the variance) as confidence ellipsoids around the samples [68]. The PCoA was visualized with EMPeror [69].

The sequence data are available at the NCBI short read repository under the accession number SRR6080480.

## Results

### Microscopy analysis

The morphological analyses of fungal spores resulted in the identification of 22 genera (S1 Table). The morphological and molecular identification fully corresponded for the genera *Alternaria*, *Cladosporium*, *Stemphylium* and *Torula* in all sites. Spores of the genera *Leptosphaerulina*, *Oidium*, *Peronospora*, *Pithomyces* and *Polythrincium* (single spores found in Marche and Veneto), and of the lichen genus *Caloplaca* were observed on the tapes but corresponding sequence were not recovered by the molecular analysis (S1 and S2 Tables). Also, *Caloplaca*, *Leptosphaerulina* and *Oidium* were detected in only one of the four sites, precisely in Marche, FVG and Umbria, respectively.

### Sequencing and data analysis

A total of 328,929 raw reads were generated, 176,054 passed the quality filter and had an average length of 385 bp. After ITS2 extraction and chimera checking a total of 152,418 reads, ranging from 496 to 6,356 reads per sample, were retained and represented the final dataset used for the taxonomic assignment and the statistical analyses (S3 Table).

Rarefaction curves showed a large variation in the total number of OTUs among samples. The curves did not reach saturation (S1 Fig), suggesting that an increased sequencing depth would detect additional OTUs.

### Comparison within and among sites

Alpha and beta diversity of samples were estimated from the rarefied dataset with a minimum value of 1,284 reads. Two samples from Veneto, V5 and V7, resulted in only 496 and 558 reads, respectively, and were therefore excluded from the further analyses (S3 Table).

**Fig 2:**
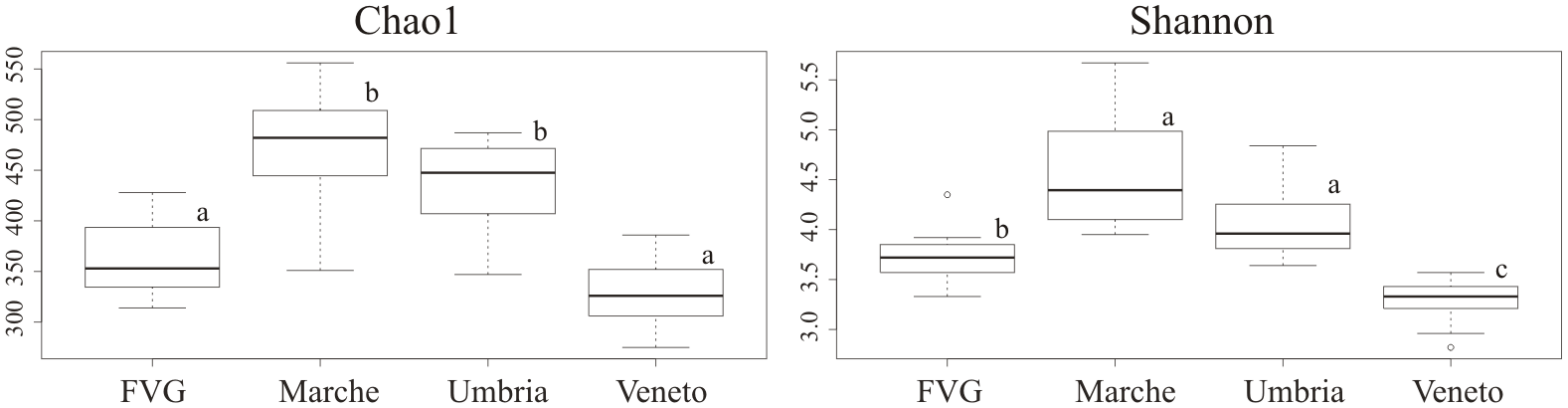
Box plots of Chao1 and Shannon diversity indices estimated for each site. Significant differences among sites were calculated with ANOVA and Duncan’s new multiple range test and are indicated by different letters a-c (p-value < 0.05).

The alpha diversity was estimated using Chao1 and Shannon diversity indices (S3 Table). A significant difference (with ANOVA and Duncan’s new multiple range test p-value <0.05) was recorded in comparing the two indices between sites (Fig 2, S3 Table). Both Chao1 and Shannon indices were significantly higher (with ANOVA and Duncan’s new multiple range test p < 0.05) for the two sites from the Central Italy, Marche and Umbria, than with the two sites from the NE Italy, FVG and Veneto. Veneto showed the lowest diversity values (Fig 2, S3 Table). At genus level, 195 and 194 taxa were found for Marche and Umbria, respectively, while only 113 and 97 were reported for FVG and Veneto, respectively.

**Fig 3:**
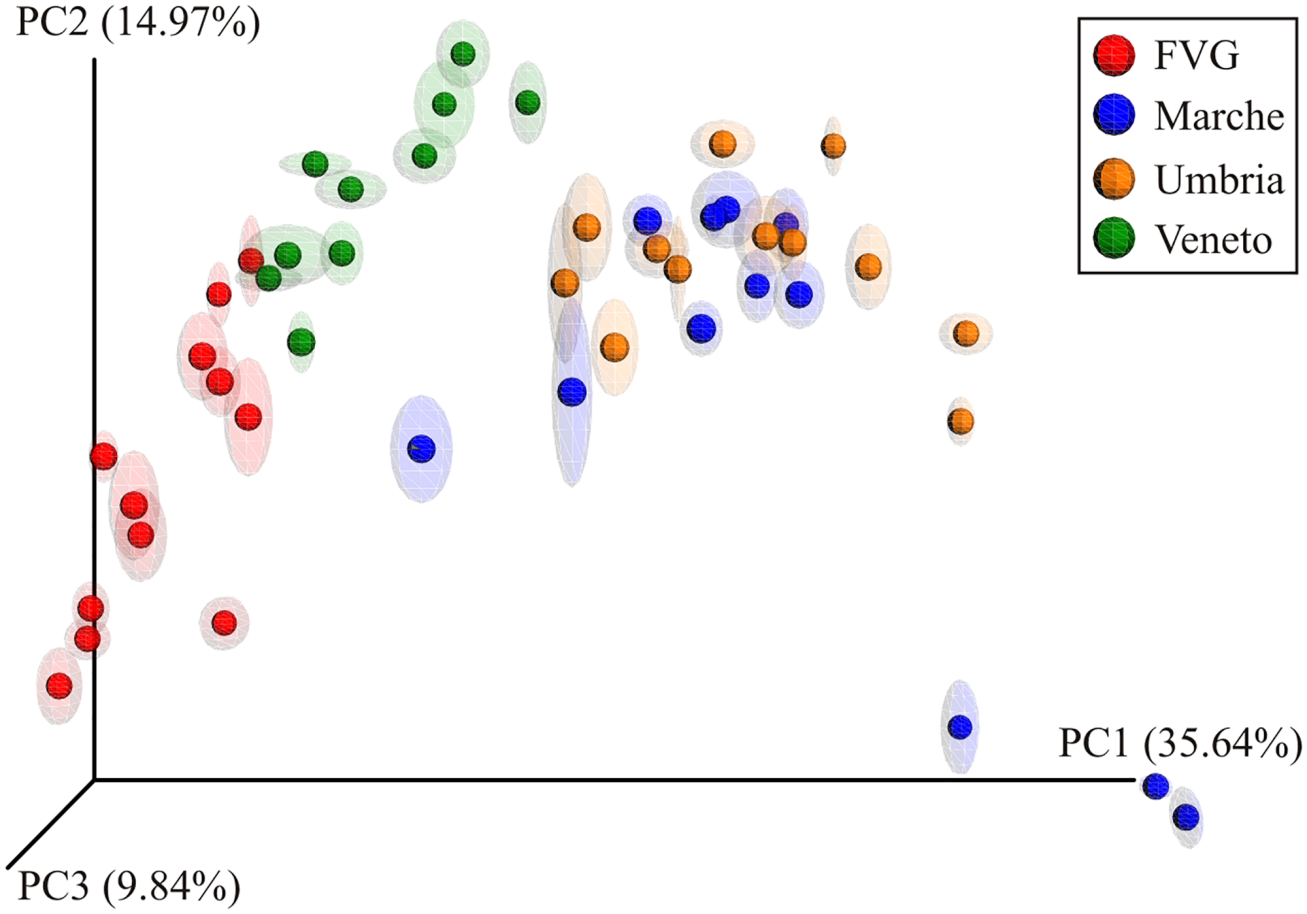
Jackknifed principal coordinate analysis (PCoA) plot of Bray-Curtis distances between the samples of the four sites. Ellipsoids show the statistical confidence of the analysis.

The beta diversity was assessed from Bray-Curtis distance matrices and presented with PCoA plot. The maximum percentage of variation explained by the single PC1 axis was 35.64% (Fig 3). The samples are grouped mostly according to their geography: samples from NE Italy (FVG and Veneto) are well separated between each other and also from the samples from the Central Italy (Marche and Umbria), while these latter were clustered together. This is due to an uneven distribution of the taxa in the sites: about 25% of the genera (63 out of 239) detected in the dataset are present only in the sites of Central Italy. On the other hand, in NE sites we recovered a high number of reads belonging to unidentified fungi, such as uncultured fungi (about 30% and 20% in FVG and Veneto, respectively; 8% in both Marche and Umbria). In particular, in NE sites about 15% of the reads belong to an uncultured fungus isolated in air sample in (GeneBank ID KF800389).

Within the Central Italian samples, only three samples from Marche (M2, M3 and M4) distinctly segregate from the others (Fig 3), likely due to a higher presence of Phaeosphaerales and Pleosporales and a lower presence of Capnodiales OTUs (S2 Fig).

### Taxonomic composition

**Fig 4:**
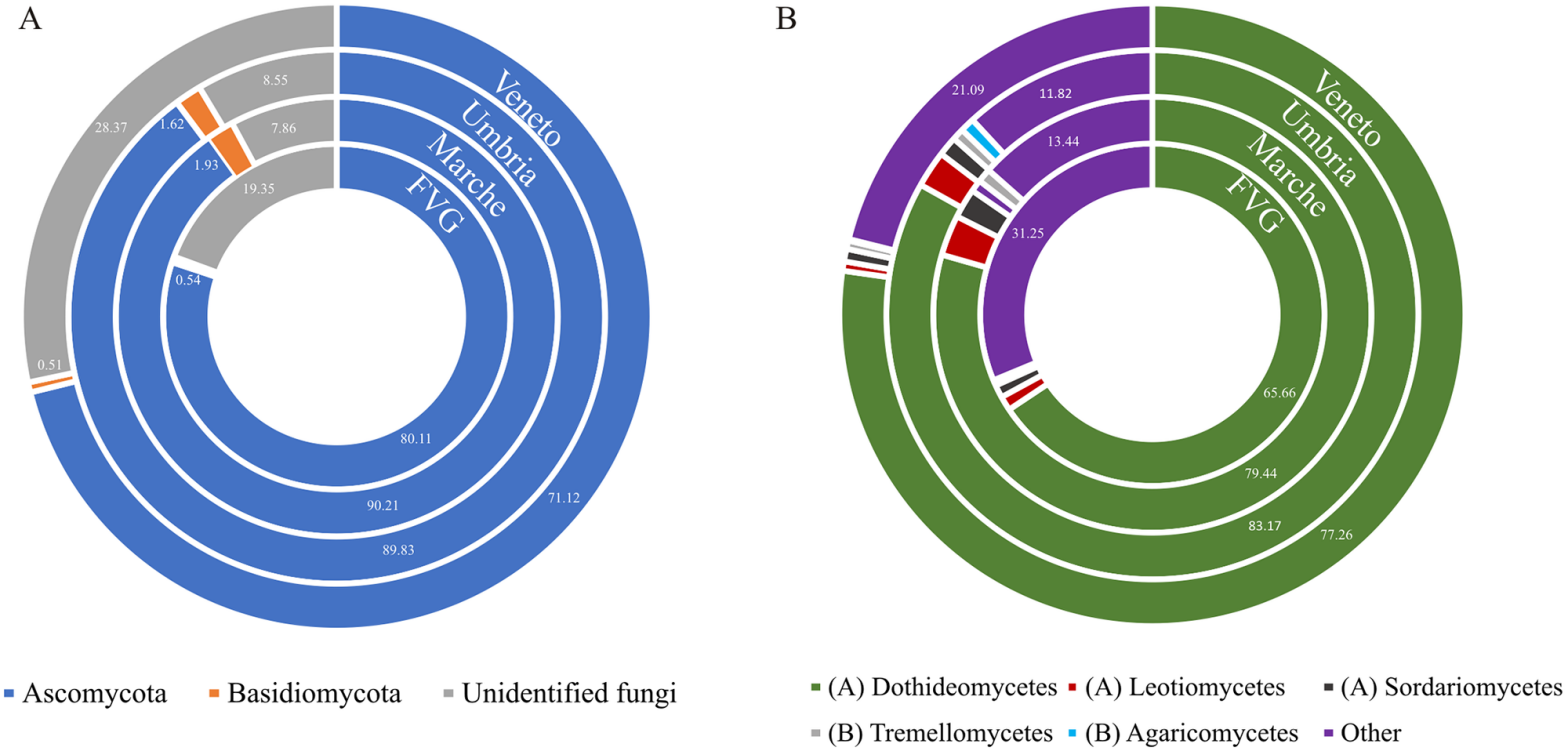
Doughnut charts showing fungal taxonomic composition at division (A) and class (B) level in the four sampling sites. Relative abundances of taxa are reported in percentage. “Unidentified fungi” comprehends Fungi sp. and fungi with no blast hit. Taxa accounting for <0.1% of reads are grouped as “other”.

The ITS2 analysis of airborne fungi allowed a taxonomic assignment for more than the 99% of the reads by clustering them to OTU at 97% similarity (S2 Fig). At division level, in all sampling sites the vast majority of reads belonged to ascomycetes (Fig 4A). However, between the different sites the distribution at division level is not homogenous. The two sites in NE Italy (FVG and Veneto) present a lower proportion of ascomycetes (71.12 and 80.11%, respectively) but a higher proportion of reads that could be assigned only at kingdom level (Fungi sp. 28.2% and 19.1%, respectively, which includes environmental and uncultured fungi) than the two sites of Central Italy. These latter, Marche and Umbria, are more similar to each other in the amount of ascomycetes (90.21% and 89.83% reads, respectively) and in the amount of Fungi sp. (7.3% and 7.9%, respectively; Fig 4A). In all sites less than 1% of the reads correspond to fungi with no blast hit (0.12% in FVG, 0.93% in Marche, 0.31% in Umbria and 0.20% in Veneto). Basidiomycetes are present in very low proportions, representing less than 1% in FVG and Veneto and between 1.5–2% in Marche and Umbria. The most dominant ascomycetes class is Dothideomycetes, followed by Leotiomycetes and Sordariomycetes (Fig 4B). Basidiomycetes are represented by Tremellomycetes and Agaricomycetes (Fig 4B).

**Fig 5:**
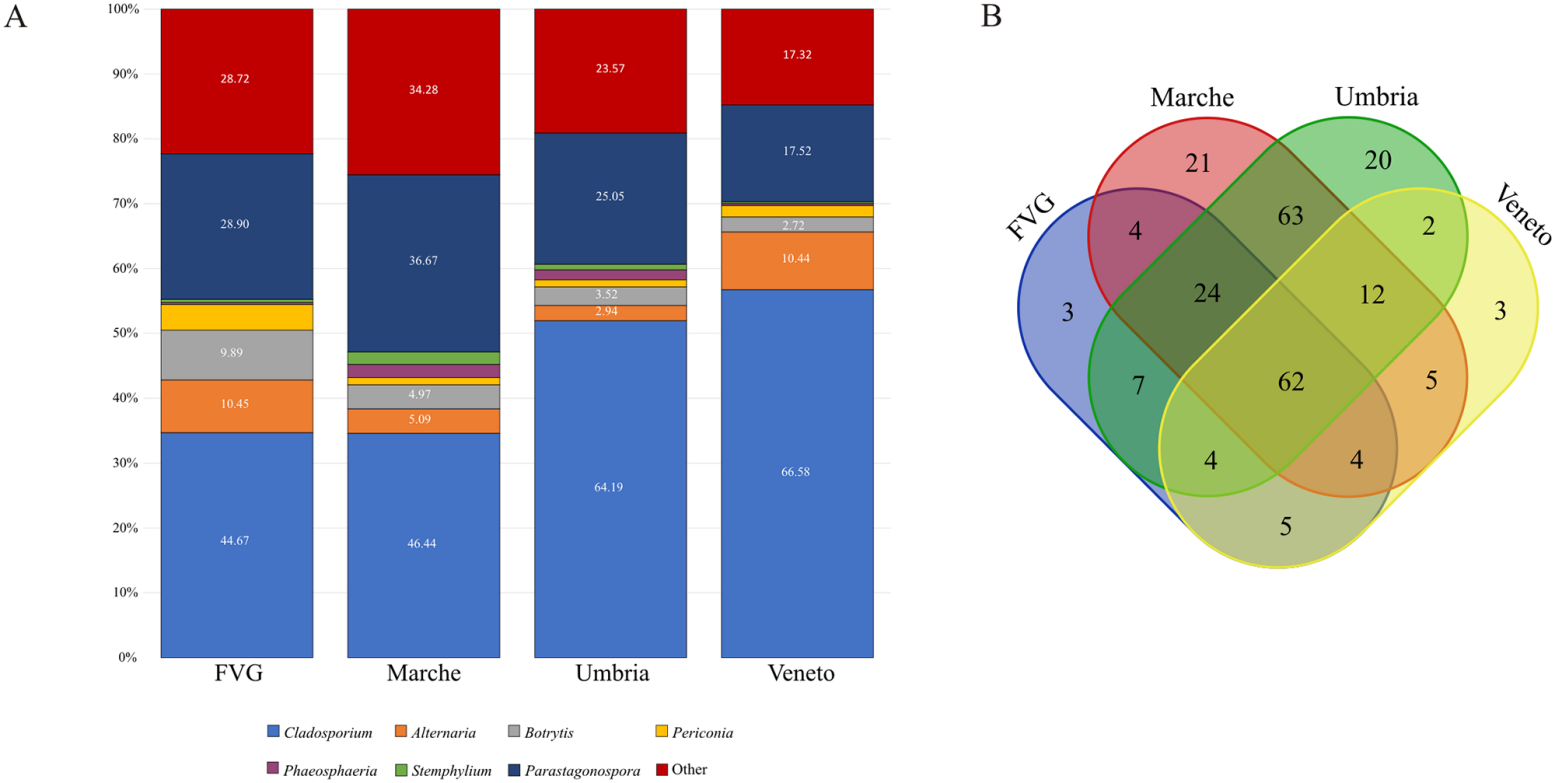
A) Bar charts showing the taxonomic composition at genus level in the four sampling sites. Abundances of taxa are reported with the percentage values of reads. Taxa accounting for <0.1% of reads are grouped as “Other”. B) Venn diagram shows the number of unique and shared taxa identified at the genus level among sites (as in S2 Table).

The order Capnodiales was the most represented order in all the sites and was followed by Pleosporales. The most represented genera in all the sites were *Cladosporium*, *Alternaria*, *Botrytis* and *Periconia* (Fig 5A).

Sixty-two taxa identified up to the genus level are shared by the four sites, comprising about 99% of the total reads, (Fig 5B; being *Cladosporium*, *Alternaria*, *Botrytis* and *Periconia* the most abundant). FVG, Marche and Umbria shared 24 taxa (being *Naevala* and *Vuilleminia* the most abundant genera, both representing >0.1% of the reads). Low represented taxa (<0.1% of the reads) are shared among two or three of the four sites (S2 Table). In each site, we also recover unique taxa (<0.1% of the reads) which were otherwise not detected in the other three sites: three for FVG and Veneto, 20 for Umbria and 21 for Marche (S2 Table).

At species level, in all four sites the most represented species was *Cladosporium herbarum* (the anamorph synonym of *Mycosphaerella tassiana*, [70–73]). In FVG, *M*. *tassiana* (5.59%) was followed by *Botrytis cinerea* (1.25%), *Alternaria eichhorniae* (0.64%), *Exserohilum oryzicola* (0.6%), *Periconia pseudobyssoides* (0.46%), *Hannaella luteola* (0.24%), *Alternaria alternata* (0.13%), *Bipolaris sorokiniana* and *Aspergillus intermedius* (0.12% each). In Marche, *C*. *herbarum* (14.35%) was followed by *B*. *cinerea* (1.55%), *Stemphylium herbarum* (0.81%), *Phaeosphaeria juncophila* (0.39%), *A*. *eichhorniae* (0.39%), *Angustimassarina acerina* (0.37%), *Lanzia echinophila* (0.32%), *Lophiostoma macrostomum* (0.27%). In Umbria, *C*. *herbarum* (18.71%) was followed by *B*. *cinerea* (1.03%), *Parastagonospora avenae* (0.43%), *P*. *juncophila* (0.41%), *L*. *echinophila* (0.33%), *A*. *eichhorniae* (0.31%), *S*. *herbarum* (0.31%), *L*. *macrostomum* (0.22% each). In Veneto, *C*. *herbarum* (9.46%) was followed by *A*. *eichhorniae* (0.84%), *B*. *cinerea* (0.39%), *H*. *luteola* (0.27%), *Periconia pseudobyssoides* (0.22%), *Aspergillus intermedius* (0.18%), *A*. *alternata* (0.14%).

### Meteorological and land use data

Values recorded in the four sites for precipitations, air temperatures, air humidity and wind speed during the sampling period were quite similar and are reported in S4 Table and showed in S3 Fig.

Land use information of the four sites for a 50 Km buffer (artificial surfaces, agricultural areas, forest and semi natural areas, wetlands and water bodies) are reported in S5 Table and showed in S4 Fig. North-eastern sites present a higher proportion of artificial surfaces and a lower proportion of forest and semi natural areas (8.87% and 28.49% in FVG, 11.25% and 22.80% in Veneto, respectively) than the site in Central Italy (3.54% and 38.60% in Marche, 2.70% and 45.20% in Umbria, respectively).

## Discussion

### Fungal diversity in airborne samples: Molecular vs morphological analyses

In this study we characterized the taxonomic composition of airborne fungi across four Italian sites using the ITS2 region as barcode and the Ion Torrent sequencing platform. The two sites from Central Italy, Marche and Umbria, showed higher species richness and diversity than the two sites in NE Italy, FVG and Veneto. Beta diversity analyses group samples on the base of their geographic location, as a subtle, different taxonomic composition was recovered between the sites. Alternatively, the fungal composition during the two sampling weeks remains rather constant within each site.

Spatial contexts (such as pedology, land use and vegetation) determine the availability of substrates and plant hosts on which certain fungi can develop and spread [74]. In this study differences in land use among the sites seem to partially explain the taxonomic diversity recovered. The higher presence of forests and semi-natural areas in the sites of Central Italy than in the NE sites, might represent the reason of a higher fungal diversity. Also, the range of dispersion that spores reach is strongly influenced by the characteristics of the landscape (whether this is natural or anthropogenic modified) at both regional and local scales [75]. The presence and distribution of fungal communities—and therefore their spores- are influenced by climatic ([76, 77] and reference therein) and meteorological conditions as well [78–81]. Mean air temperature, relative humidity and wind speed are known to be factors shaping the spore distribution of *Alternaria*, *Cladosporium*, *Drechslera*-type, *Epicoccum* and *Torula* [82]. However, here, we do not correlate the overall diversity observed with the meteorological parameters, as the recorded meteorological data were rather constant among the sites and were collected over a too short period (two weeks) to be reliably generalized in a broader context.

The main classes of fungi detected by the ITS2 barcode belonged to ascomycetes, whereas basidiomycetes were present in very low proportions. The low proportion of basidiomycetes might be impaired by multiple factors. About 15% of the reads could not be identified; these reads might indeed hide further asco- or basidiomycetes taxa as well. In outdoor airborne samples the amounts of the two fungal divisions was often reported to vary during the year, but it can also highly differ within shorter periods of time (i.e. daily [14, 50]), making the comparison of different surveys difficult. Notwithstanding this bias, the low proportion of recovered basidiomycetes could be attributed to spore dimensions, dispersal ability, weather conditions (as humidity, precipitations), seasonality, availability of substrate or geographical factors. Previous studies report that ascomycetes are more common than the basidiomycetes during dry days [14, 83]. Another study based on ITS sequencing of airborne fungi captured throughout a year reported that ascomycetes were prevailing (>90%) among larger particles (>9 μm), while the opposite trend was recorded for smaller particles (<3 μm) [24]. Among spores, ascomycetes are more often reported in higher abundances than basidiomycetes. For example, Fierer et al. [84] reported as much as 97% of ascomycetes using cloning and sequencing of the universal nuclear marker SSU, while Yang et al. [18] reported abundances reaching over 90% when sequencing fungal ITS1 in a study where haze and non-haze days in Beijing were analyzed based on the particulate matters fraction (PMs). The selection of the barcode primers can also affect the detection of certain taxa [85, 86]; it has been observed that ITS1 barcode generally captures a higher proportion of basidiomycetes than of ascomycetes [51, 87, 88].

#### The most widespread taxa

The genera *Cladosporium*, *Alternaria*, *Epicoccum* and *Stemphylium* were represented by the highest read percentages and were abundantly found in the samples inspected at the microscope as well. These genera have their peak of spore dispersal at late summer and early autumn [6, 89, 90], and indeed their spores were spread all over the collecting tapes. These fungi are also known to be among the most common causes of allergies [91, 92].

The sequencing results are characterized by a relatively high percentage of reads here referred as Fungi sp. (about 7% for central and 25% for NE sites, respectively). These correspond to about 100 OTUs which blasted in NCBI mainly as “Fungal sp.” or “Uncultured fungus” derived from other air surveys (i.e. FJ820545, KP724985, KF800623), confirming the importance of further investigation on this pool of still unknown, ecological components.

#### Peculiar taxa

Interestingly, our molecular analyses catch the presence of fungal taxa that are not expected in urban areas and are peculiar because of their life styles, being these lichenized and rock-inhabiting fungi. Indeed, we report the presence of eight lichen genera (*Caloplaca*, *Cladonia*, *Flavoparmelia*, *Lecidella*, *Physcia*, *Hyperphyscia*, *Rinodina*, *Umbilicaria*) of which the spores of only the genus *Caloplaca* were identified during the morphological inspections of the samples. The majority of the detected taxa are epiphytic lichens commonly distributed in Italy, and can occur also in urban environment if these are not highly polluted. The only exception is the genus *Umbilicaria* which comprises of only epilithic species occurring in montane and alpine environments. To the best of our knowledge, this is the first report of the detection of lichen spores and lichen sequence data in airborne samples.

Also, the genus *Schizoxylon* is one of the 70 genera shared by Marche and Umbria. Interestingly this fungus is known to be both saprotrophic and optionally lichenized [93–95] and has been reported only for Scandinavian countries so far [94]. Its presence in our dataset lets us speculate on its possible distribution in the Mediterranean region but further researches are necessary to support this hypothesis.

Surprisingly, rock inhabiting fungi (RIF) were identified as well, though in low amounts. Some of them belong to widespread genera, such as *Knufia*, while other sequences correspond to extremophilic genera, such as the *Friedmanniomyces* [96]. It is likely that either their presence in the airborne samples is due to long distance dispersal, or that their identities was corresponded to closely related taxa which colonize rock surfaces in, or close to, the urban environments. The distribution of epilithic and endolithic RIF assemblages is still poorly known, and whether they would be repeatedly recovered in airborne samples should be investigated further.

#### Microscopy vs molecular analysis

The lack of detection of certain taxa by molecular analysis could be due to different reasons: the presence of a primer bias towards those taxa (the more likely case in this study), the fact that low represented sequences (*i*.*e*. singletons) are removed from the dataset, or the lack of representative sequences in public databases, with which our data can be compared and referenced to. Moreover, in case of taxa with particular difficult morphology, the identification can remain ambiguous.

As some taxa were identified only by one of the two techniques, both approaches are still to be implemented to gain an integrated and comprehensive overview of fungal diversity in airborne samples. On one hand, microscopy analyses allow to perform quantitative estimation of the samples (particles/m^3^), is a less elaborate method and keeps laboratory costs low. On the other hand, it renders possible only a shallow taxonomical identification, and this can be affected by the degree of expertise and the specialization of the operator. Molecular tools such as DNA metabarcoding, on the contrary, are independent from the operator expertise and allow to detect a much higher taxonomic biodiversity within the samples. However, costs and time for the analyses are higher and results might be biased by the preferential amplification of certain sequences or lack of representative, reference sequences in public databases.

### Fungal pathogens and invasive alien species (IAS)

Both microscopy and molecular analyses detected in all the four sites fungal taxa which are well known plant (e.g. *Botrytis*, *Bipolaris*, *Periconia*, *Phaeosphaeria*, *Parastagonospora*, *Ramularia*, *Stemphylium*) and animal pathogens (e.g. *Acremonium*, *Candida*, *Cryptococcus*, *Torula*) or represent alien species. Within the most detected fungal order Capnodiales (Ascomycota, Dothideomycetes) the main genera are the cause of many human allergenic diseases, e.g. *Alternaria*, *Aspergillus*, *Cladosporium*, *Epicoccum* and *Exserohilum*. Species of the genus *Alternaria* are saprotroph, present a worldwide distribution and are commonly found in diverse habitats [97]. *Alternaria* species can be found also on human skin [98] and seems to be the most significant fungal allergen for people [99].

The genus *Aspergillus* comprehends species that are highly aerobic and commonly grow as molds on the external surface of diverse substrates. More than half of known *Aspergillus* species are identified only in their anamorphic state; the majority of the species are clinically and commercially important (such as human pathogens on skin or fungi used in fermentation processes), others are sources of natural products implemented in the treatment of many human diseases [100].

*Cladosporium* is a worldwide spread genus, it occurs on different substrates and includes species with diverse lifestyles [101, 102]. It is abundantly found on dead leaves of herbaceous and woody plants and has frequently been isolated from air [103], where it usually represents the most abundant fungus both in indoor and outdoor environments [99]. Similarly, species of the genera *Epicoccum* and *Stemphylium* are saprophyte and widely distributed; *Stemphylium* is also reported as weak parasite and pathogen in plants, including crops [104].

The genera which were detected only by microscopy analyses are *Leptosphaerulina*, *Oidium*, *Peronospora*, *Pithomyces* and *Polythrincium*. *Leptosphaerulina* has been classified as a saprophyte and pathogen of turfgrasses [105]. *Oidium* is an obligate, biotrophic, powdery mildew genus [106]. *Peronospora* is a genus of oomycetes considered to be the largest downy mildew genus [107]. Both *Oidium* and *Peronospora* cause significant economic impact in crops and ornamental plants [108]. *Pithomyces* is a large genus in the order Pleosporales; it commonly colonize plants, dead leaves and stems [109]. Different studies have highlighted its presence in air of indoor environment where asthmatic patients were hosted [110]. *Polythrincium*, belonging to Capnodiales, is an obligate biotrophic and is the pathogen causing the sooty/black blotch of clover [111].

Invasive alien species (IAS) are recognized as a major threat of diverse ecosystems [112]. Due to their inconspicuous nature and the fact that they are still poorly studied also in terms of bio- and phylogeography, fungal reports in IAS databases is still very scarce, with the exception of few important plants and animal pathogen [113]. The DAISIE European Invasive Alien Species Gateway (http://www.europe-aliens.org/) lists about 40 alien fungal species for Italy. Among these, *Discula destructiva* was sequenced from the sampling site in Umbria. This fungus is a pathogenic, causal agent of the dogwood anthracnose, which is one of the major diseases affecting *Cornus* tree species [114]. First observed in North America [115], the disease has been reported also in Germany since 2002 [116] and in Italy since 2003 [117].

### HTS technology for the study of airborne fungi

In our survey, species accumulation curves did not reach saturation, indicating an even more remarkable richness and diversity of taxa. A more exhaustive sampling could be obtained if the sequencing depth would be increased, for example by using a larger PGM chips (such as 316^™^) Another possibility would be to use other HTS approaches that allow the sequencing of the whole ITS fragment.

The application of HTS technologies is nowadays among the new, standard approaches for environmental studies. Despite the great advantages offered by HTS, e.g. the high taxonomic resolution, reproducibility and short processing time [118], DNA metabarcoding is still affected by some pitfalls. Among them, the possibility to quantify the abundance of the taxa with higher accuracy (stochasticity of the PCR amplifications and also sequencing results are semi-quantitative at best) and primer bias impair sequencing results at the most [118–120]. Further, the underestimation of species diversity is unpredictable in several fungal taxa, although primers have been designed *ad hoc* for certain groups [120–122]. The low proportion of basidiomycetes (see previous section) detected in this survey might be attributed to the selected primers (ITS3/ITS4), as they were shown to preferentially amplify ascomycetes [51, 87, 88]. The cause of this could be that the ITS2 amplicon is longer in basidiomycetes than in ascomycetes (about 30–50 bp longer [88]) and therefore the selected primer pair may preferentially amplify the shorter ITS2 in the ascomycetes [87, 88], explaining the higher proportion of ascomycetes reads in our dataset. As the application of HTS to fungal communities studies is increasing, there is a general need to develop primers that minimize the taxonomic biases so far persisting [86, 123].

The identities of the generated sequences are still hardly comparable with reference databases and this represent a drawback in such surveys. We expect that the establishment of site-specific reference databases would implement in the future the identification of airborne fungal particles and further improve the air monitoring.

## Conclusions

Intraspecific morphological variation, low quantity and lack of distinctive morphological characters have been the major constraints for microscopy identification of fungal spores in airborne samples. In the present study we have showed that the great number of taxa identified with DNA metabarcoding is ten-fold higher than the one identified by microscopy analyses (238 vs. 22 genera). We found correspondence between morphological and molecular analyses and provide a much more accurate determination of the taxa in comparison with the traditional morphological inspections.

This strengthens the perception that HTS analyses are tools of key importance to increase the sensitivity of air biomonitoring and our knowledge on airborne fungal diversity. The standardization of HTS techniques in aerobiology will make the monitoring of pathogenic fungal agents and their distribution affordable in shorter time and with higher reliability. The prompt identification of new or potential allergenic substances from plant and fungal tissues, as well as invasive species, is essential for an effective prevention and management of diverse environments. A long-scale monitoring extended on a wider geographic area in Italy is taking into account seasonal variation and meteorological conditions. Further, the development of regional database for airborne fungi and the ongoing implementation of existing worldwide fungal databases [124, 125] represents a reliable assessment for the identity of new sequence data.

## Supporting information

S1 Fig Rarefaction curves of the complete dataset. (TIF)

S2 Fig Bar charts showing the taxonomic composition up to class level in the 48 samples. Abundances of taxa are reported with the percentage values of reads. Taxa accounting for <0.1% of reads are grouped as “Other”.
(TIF)

S3 Fig Metereological parameters daily recorded in the four sites during the sampling period (September 2017). (TIF)

S4 Fig Land use (expressed in percentage) according to CLC12 in 50 km buffers around each of the four sampling sites. (TIF)

S1 Table List of the taxa recovered by microscopy analysis in the sampling sites. For each taxon, presence (√) or absence (-) in each sampling sites is shown for both microscopy (micr) and amplicon sequencing (DNA).
(PDF)

S2 Table List and presence of taxa down to genus level in the Venn diagram of Fig 5B. The genera represented by more than 0.1% of the reads are highlighted in bold.
(PDF)

S3 Table Summary of sequencing data and diversity estimation. (PDF)

S4 Table Metereological parameters daily recorded in the four sites during the sampling period (September 2016). (PDF)

S5 Table Land use according to CLC12 in 50 km buffers around each of the four sampling sites. (PDF)
