# Supplementary figures and images for: DNA metabarcoding uncovers fungal diversity of mixed airborne samples in Italy
Source: PLoS One. 2018 Mar 20;13(3):e0194489. doi: 10.1371/journal.pone.0194489 (PMC5860773; doi:10.1371/journal.pone.0194489)

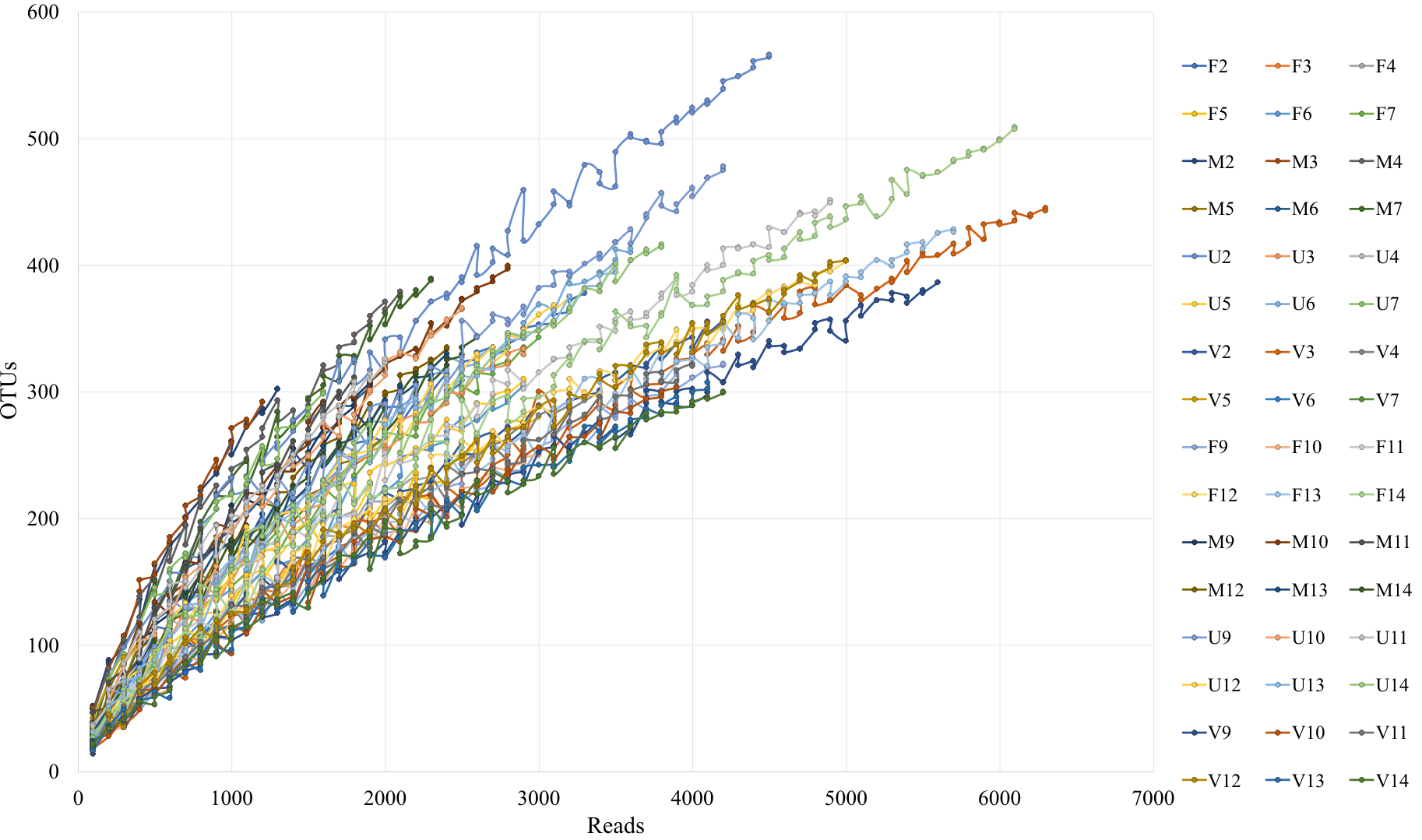

Supplement: S1 Fig — (TIF) [file pone.0194489.s001.tif]

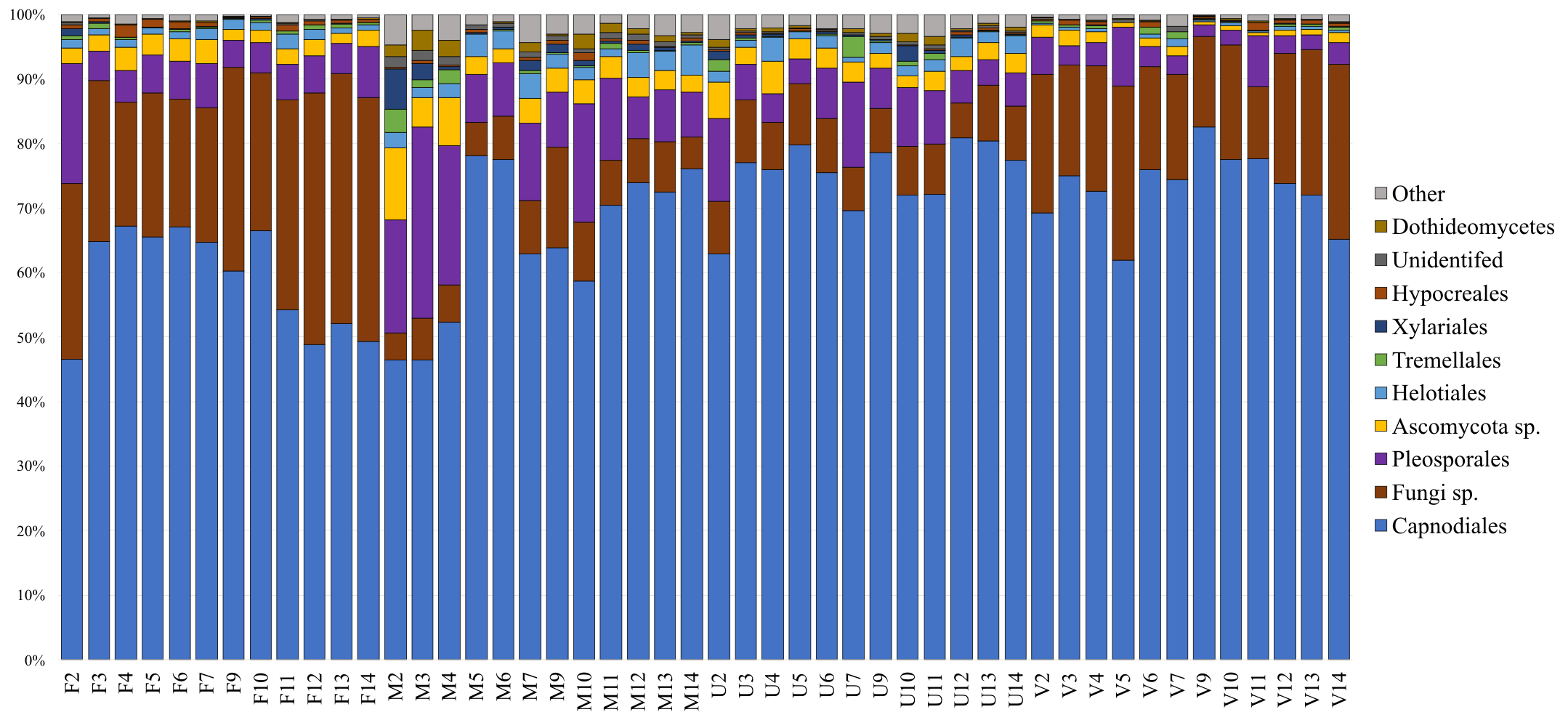

Supplement: S2 Fig — Abundances of taxa are reported with the percentage values of reads. Taxa accounting for <0.1% of reads are grouped as “Other”. (TIF) [file pone.0194489.s002.tif]

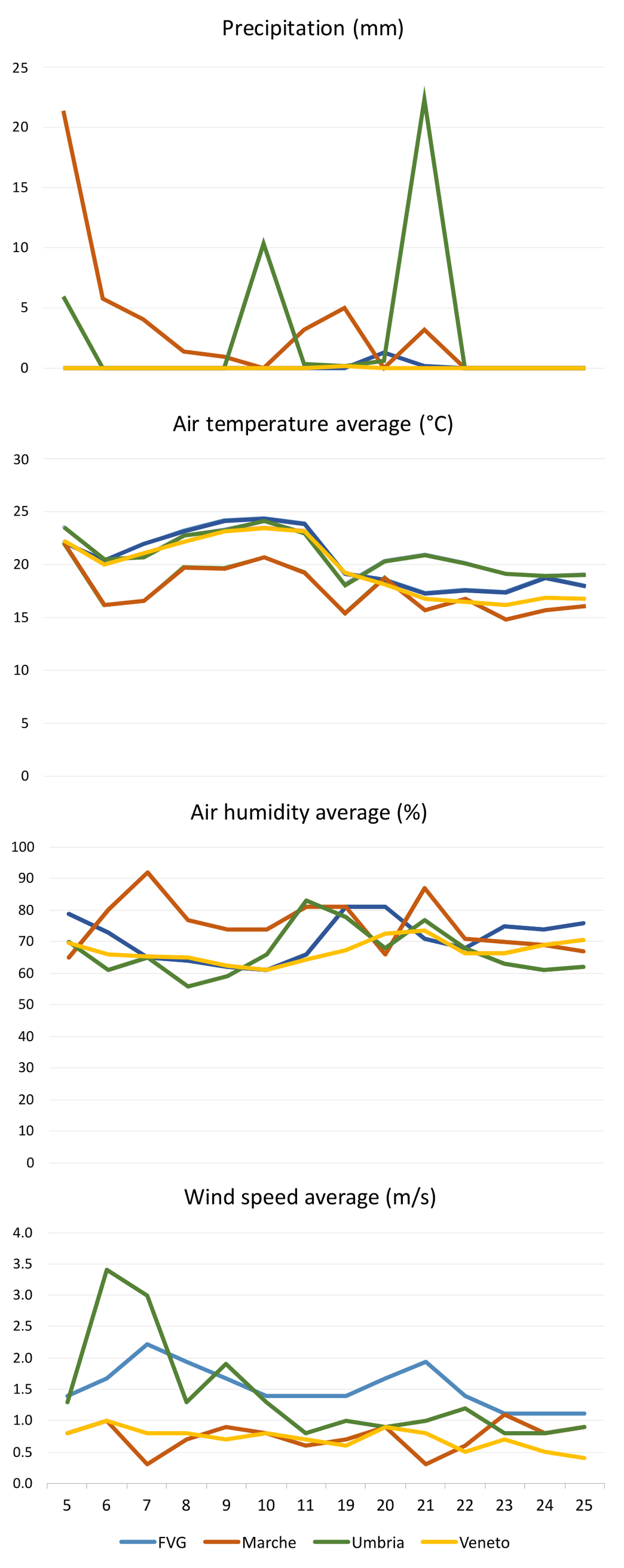

Supplement: S3 Fig — (TIF) [file pone.0194489.s003.tif]

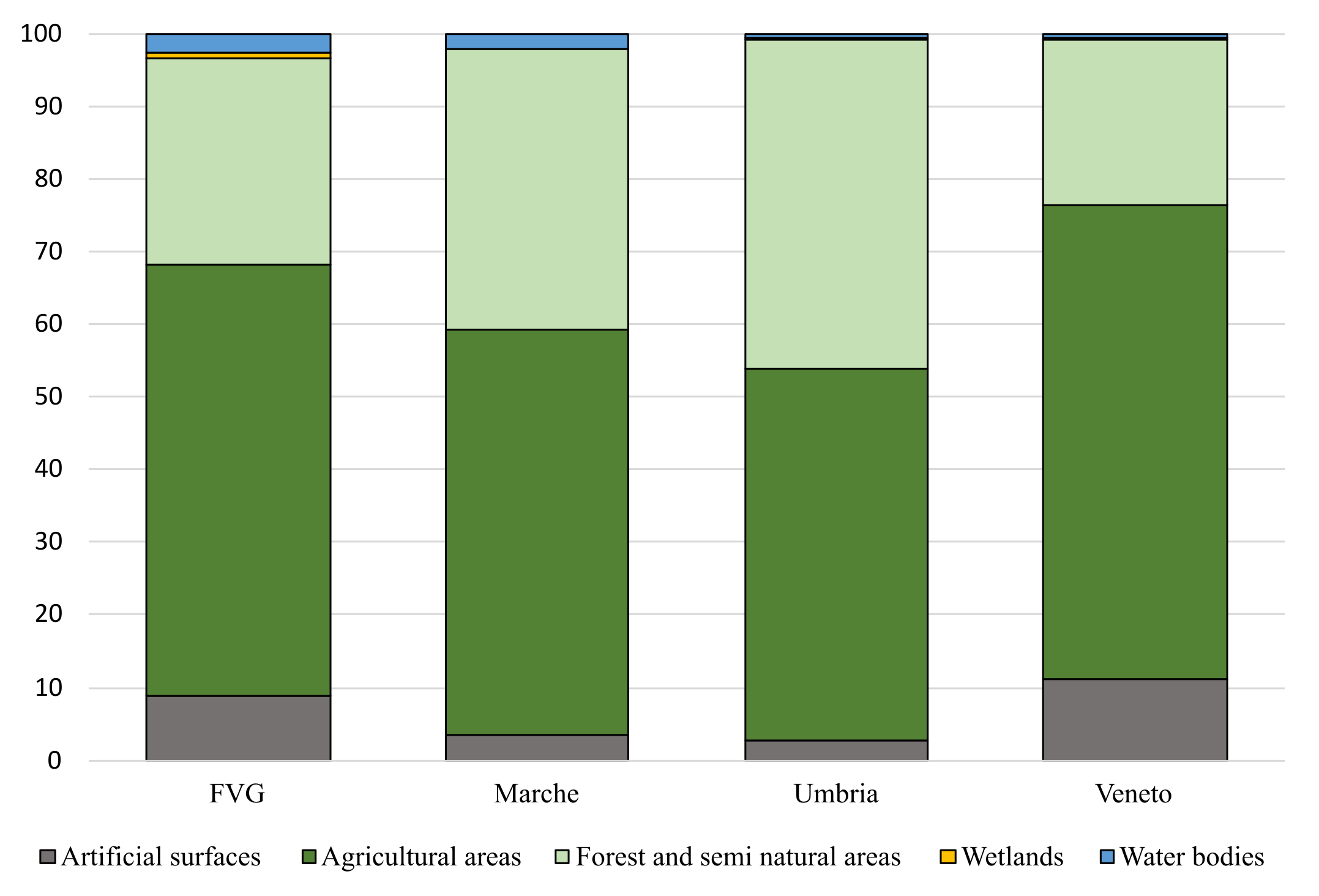

Supplement: S4 Fig — (TIF) [file pone.0194489.s004.tif]
