# Supplementary material for: DNA metabarcoding uncovers fungal diversity of mixed airborne samples in Italy
Source: PLoS One. 2018 Mar 20;13(3):e0194489. doi: 10.1371/journal.pone.0194489 (PMC5860773; doi:10.1371/journal.pone.0194489)
Supplement: S1 Table — For each taxon, presence (√) or absence (-) in each sampling sites is shown for both microscopy (micr) and amplicon sequencing (DNA). (PDF) [file pone.0194489.s005.pdf]

S1 Table. List of the taxa recovered by microscopy analysis in the sampling sites. For each taxon, presence (√) or absence (-) in each sampling sites is shown for both microscopy (micr) and amplicon sequencing (DNA).

	FVG		Marche		Umbria		Veneto	
	Micr	DNA	Micr	DNA	Micr	DNA	Micr	DNA
Alternaria	√	√	√	√	√	√	√	√
Amphisphaeria	-	√	√	√	-	-	-	-
Bipolaris	√	√	-	√	√	√	√	√
Caloplaca	-	-	√	-	-	-	-	-
Cladosporium	√	√	√	√	√	√	√	√
Curvularia	√	√	-	√	-	√	√	√
Drechslera	√	√	-	-	√	√	√	-
Epicoccum	√	√	√	√	√	√	√	-
Exserohilum	√	√	-	√	√	√	√	√
Keissleriella	-	√	-	√	-	√	√	√
Leptosphaeria	√	√	√	√	√	√	-	√
Leptosphaerulina	√	-	-	-	-	-	-	-
Lophiostoma	√	√	√	√	√	√	-	√
Massarina	-	√	√	√	√	√	√	√
Oidium	-	-	-	-	√	-	-	-
Periconia	√	√	-	√	-	√	√	√
Peronospora	√	-	√	-	-	-	√	-
Pithomyces	√	-	-	-	-	-	√	-
Pleospora	√	√	√	√	-	√	-	√
Polythrincium	-	-	√	-	-	-	√	-
Stemphylium	√	√	√	√	√	√	√	√
Torula	√	√	√	√	√	√	√	√
