# Supplementary material for: DNA metabarcoding uncovers fungal diversity of mixed airborne samples in Italy
Source: PLoS One. 2018 Mar 20;13(3):e0194489. doi: 10.1371/journal.pone.0194489 (PMC5860773; doi:10.1371/journal.pone.0194489)
Supplement: S2 Table — The genera represented by more than 0.1% of the reads are highlighted in bold. (PDF) [file pone.0194489.s006.pdf]

S2 Table. List and presence of genera in the Venn diagram of Fig. 5B. The genera represented by more than 0.1% of the reads are highlighted in bold.

FVG	Marche	Umbria	Veneto	Nr. of shared taxa	Taxa (genus level)
√	√	√	√	62	*Acremonium*, ***Alternaria***, ***Angustimassarina***, *Arthrinium*, *Ascochyta*, ***Aspergillus***, *Aureobasidium*, *Biatriospora*, *Bipolaris*, ***Botrytis***, ***Bullera***, *Candida*, ***Cercospora***,*Chalastospora*, *Ciboria*, ***Cladosporium***,*Coprinellus*,*Cryptococcus*,*Curvularia*,*Dendryphion*, *Didymella*, ***Dioszegia***, ***Exserohilum***, *Fusarium*, *Ganoderma*, *Gibberella*, ***Hannaella***, *Hansfordia*, *Hymenoscyphus*, *Hyphodermella*, *Hyphodontia*, ***Hypoxylon***, *Keissleriella*, L***anzia***, *Leptosphaeria*, *Leptospora*, ***Lophiostoma***, *Massarina*, *Monographella*, ***Mycosphaerella***, *Myrmecridium*, *Nigrospora*, *Paraconiothyrium*, ***Parastagonospora***, ***Passalora***, *Penicillium*, ***Periconia***, ***Phaeosphaeria***, ***Phoma***, *Pleospora*, *Podosphaera*,*Pseudodidymosphaeria*, *Sawadaea*, *Septoria*, *Setophaeosphaeria*, *Sordaria*, ***Stagonospora***, ***Stemphylium***, *Teichospora*, *Torula*, ***Xenobotryosphaeria***, *Xylaria*
√	√	√		24	*Bulleribasidium*, *Cladophialophora*, *Comoclathris*, *Coniothyrium*, *Epicoccum*, *Eutypa*, *Extremus*, *Lachnum*, *Lentithecium*, ***Naevala***, *Neoascochyta*, *Neodevriesia*, *Neofusicoccum*, *Neokalmusia*, *Phialocephala*, *Plectania*, *Pleurophragmium*, *Pyrenochaetopsis*, *Rinodina*, *Sphaerellopsis*, *Sporobolomyces*, *Truncatella*, ***Vuilleminia***, *Xylodon*
	√	√	√	12	*Basidiodendron*, *Coriolopsis*, *Dactylaria*, *Incrucipulum*, *Lophodermium*, *Preussia*, *Pyrenophora*, *Rachicladosporium*, *Rutstroemia*, *Schizopora*, *Scirrhia*, *Umbilicaria*
√		√	√	4	*Endoconidioma*, *Peniophorella*, *Phaeodactylium*, *Pilidium*
√	√		√	4	*Cladonia*, *Coprinopsis*, *Golovinomyces*, *Physisporinus*
√	√			4	*Annulohypoxylon*, *Blumeria*, *Datronia*, *Uwebraunia*
	√	√		63	*Acicuseptoria*, *Auricularia*, *Botryosphaeria*, *Byssomerulius*, *Cadophora*, *Capnobotryella*, *Chalara*, *Ciborinia*, *Clathrosphaerina*, *Cryptosphaeria*, *Cryptovalsa*, *Cyphellophora*, *Diaporthe*, *Diatrypella*, *Dichomitus*, *Discosia*, *Dissoconium*, *Exidia*, *Exidiopsis*, *Friedmanniomyces*, *Fuscoporia*, *Glarea*, *Gloeophyllum*, *Gnomoniopsis*, *Graphostroma*, *Hypholoma*, *Knufia*, *Laetiporus*, *Lenzites*, *Melanconium*, *Mollisia*, *Mycoacia*, *Nemania*, *Neocladophialophora*, *Neosetophoma*, *Noosia*, *Penidiella*, *Peniophora*, *Perenniporia*, *Pezicula*, *Phaeosphaeriopsis*, *Phlebia*, *Phlebiella*, *Physcia*, *Plenodomus*, *Populocrescentia*, *Pringsheimia*, *Protodontia*, *Ramularia*, *Resupinatus*, *Reticulascus*, *Schizoxylon*, *Sclerotinia*, *Seimatosporium*, *Sistotremastrum*, *Spiroplana*, *Toxicocladosporium*, *Tremella*, *Trichopezizella*, *Valdensinia*, *Volucrispora*, *Xenasma*, *Xeropilidium*
		√	√	2	*Clohesyomyces*, *Hysterium*
√			√	5	*Hyphopichia*, *Paraphoma*, *Pichia*, *Setosphaeria*, *Xenodidymella*
√		√		7	*Adisciso*, *Camarosporium*, *Drechslera*, *Fomes*, *Fomitopsis*, *Neophaeomoniella*, *Phaeomollisia*
	√		√	5	*Abortiporus*, *Catenulostroma*, *Menispora*, *Radulidium*, *Sclerostagonospora*

√				3	*Colletotrichum, Lecidella, Paramycosphaerella*
	√			21	*Allophaeosphaeria, Austroafricana, Calycina, Cylindrium, Derxomyces, Dinemasporium, Eutypella, Hirsutella, Inocybe, Leuconeurospora, Mycena, Periconiella, Phialemoniopsis, Physalospora, Pluteus, Psathyrella, Roseodiscus, Sydowia, Torrendiella, Trametes, Trichopeziza*
		√		20	*Ampelomyces, Candelariella, Celosporium, Ceriporia, Cordyceps, Discostroma, Discula, Flavoparmelia, Gregarithecium, Helicoma, Hexagonia, Hyperphyscia, Letendraea, Neurospora, Pestalotiopsis, Phacidium, Podospora, Stereum, Terana, Tetracladium*
			√	3	*Kodamaea, Lentinus. Wallemia*
