# Supplementary material for: DNA metabarcoding uncovers fungal diversity of mixed airborne samples in Italy
Source: PLoS One. 2018 Mar 20;13(3):e0194489. doi: 10.1371/journal.pone.0194489 (PMC5860773; doi:10.1371/journal.pone.0194489)
Supplement: S3 Table — (PDF) [file pone.0194489.s007.pdf]

S3 Table. Summary of sequencing data and diversity estimation.

ID	Site	Reads	OTUs	Chao1 (SD)	Shannon (SD)
F2	FVG	3,330	379	416 (174)	4.35 (0.22)
F3	FVG	2,074	212	330 (151)	3.57 (0.17)
F4	FVG	2,374	242	353 (166)	3.66 (0.17)
F5	FVG	3,076	289	337 (170)	3.7 (0.19)
F6	FVG	2,959	308	370 (168)	3.81 (0.2)
F7	FVG	3,015	343	428 (184)	3.92 (0.2)
F9	FVG	4,211	323	352 (217)	3.33 (0.17)
F10	FVG	3,066	254	314 (153)	3.42 (0.16)
F11	FVG	4,982	454	408 (188)	3.86 (0.19)
F12	FVG	5,008	403	353 (169)	3.74 (0.18)
F13	FVG	5,749	429	332 (177)	3.57 (0.17)
F14	FVG	6,151	509	379 (173)	3.84 (0.17)
M2	Marche	1,369	312	492 (151)	5.58 (0.3)
M3	Marche	1,284	306	556 (227)	5.67 (0.31)
M4	Marche	2,142	381	472 (172)	5.33 (0.3)
M5	Marche	2,025	266	351 (135)	4 (0.22)
M6	Marche	1,935	307	511 (194)	4.37 (0.23)
M7	Marche	2,303	389	507 (174)	4.64 (0.29)
M9	Marche	1,915	307	502 (187)	4.31 (0.2)
M10	Marche	2,849	401	455 (193)	4.62 (0.27)
M11	Marche	1,847	314	523 (232)	4.42 (0.24)
M12	Marche	2,446	338	432 (172)	4.11 (0.23)
M13	Marche	2,434	331	463 (197)	4.09 (0.22)
M14	Marche	2,664	346	434 (183)	3.95 (0.21)
U2	Umbria	4,549	569	467 (195)	4.68 (0.27)
U3	Umbria	2,998	342	406 (178)	3.77 (0.18)
U4	Umbria	1,886	248	347 (127)	3.93 (0.23)
U5	Umbria	2,919	311	406 (185)	3.64 (0.2)
U6	Umbria	3,671	417	408 (171)	4.01 (0.22)
U7	Umbria	1,627	301	465 (174)	4.84 (0.3)
U9	Umbria	4,270	480	483 (210)	3.85 (0.22)
U10	Umbria	2,519	367	476 (200)	4.2 (0.27)
U11	Umbria	2,047	328	487 (184)	4.31 (0.25)
U12	Umbria	3,218	376	452 (201)	3.68 (0.23)
U13	Umbria	3,585	402	443 (195)	3.99 (0.22)
U14	Umbria	3,871	420	429 (201)	3.88 (0.22)
V2	Veneto	4,278	358	343 (150)	3.57 (0.17)
V3	Veneto	6,356	447	328 (147)	3.34 (0.19)
V4	Veneto	3,435	302	352 (164)	3.48 (0.15)
V5	Veneto	496	120	/	/
V6	Veneto	1,783	183	306 (124)	3.43 (0.17)
V7	Veneto	558	80	/	/
V9	Veneto	5,681	392	386 (204)	2.82 (0.19)
V10	Veneto	3,935	304	324 (158)	2.96 (0.15)
V11	Veneto	4,086	327	320 (152)	3.27 (0.16)
V12	Veneto	5,029	406	378 (174)	3.32 (0.16)
V13	Veneto	4,192	308	305 (150)	3.21 (0.15)
V14	Veneto	4,221	301	275 (129)	3.34 (0.16)

Sample ID, sampling provenience and number of reads (after ITS2 extraction and chimera removal) are reported. The number of detected OTUs is assessed for the entire sample, values of Chao1 and Shannon diversity indices are calculated on the dataset rarefied to 1284 reads. (/) Alpha diversity is not calculated due to a number of reads lower than 1284. SD: standard deviation.
