# Supplementary material for: DNA metabarcoding uncovers fungal diversity of mixed airborne samples in Italy
Source: PLoS One. 2018 Mar 20;13(3):e0194489. doi: 10.1371/journal.pone.0194489 (PMC5860773; doi:10.1371/journal.pone.0194489)
Supplement: S4 Table — (PDF) [file pone.0194489.s008.pdf]

S4 Table. Metereological parameters daily recorded in the four sites during the sampling period (September 2016).

**FVG**	**F1**	**F2**	**F3**	**F4**	**F5**	**F6**	**F7**	**F8**	**F9**	**F10**	**F11**	**F12**	**F13**	**F14**
Precipitation (mm)	0.0	0.0	0.0	0.0	0.0	0.0	0.0	0.0	1.3	0.2	0.0	0.0	0.0	0.0
Air temperature min (°C)	18.5	15.8	13.9	15.7	16.2	17.4	17.4	13.8	16.0	14.6	12.4	10.8	13.2	11.7
Air temperature average (°C)	22.1	20.4	22.0	23.2	24.2	24.4	23.9	19.2	18.6	17.3	17.6	17.4	18.8	18.0
Air temperature max (°C)	27.5	26.2	30.2	30.8	32.1	32.2	31.2	25.8	25.8	21.6	24.0	24.0	26.1	25.6
Air humidity average (%)	79.0	73.0	65.0	64.0	62.0	61.0	66.0	81.0	81.0	71.0	68.0	75.0	74.0	76.0
Wind speed average (m/s)	1.4	1.7	2.2	1.9	1.7	1.4	1.4	1.4	1.7	1.9	1.4	1.1	1.1	1.1

**Marche**	**M1**	**M2**	**M3**	**M4**	**M5**	**M6**	**M7**	**M8**	**M9**	**M10**	**M11**	**M12**	**M13**	**M14**
Precipitation (mm)	21.4	5.8	4.1	1.4	1.0	0.0	3.2	5.0	0.0	3.2	0.0	0.0	0.0	0.0
Air temperature min (°C)	15.0	11.0	14.1	15.0	14.6	16.6	15.7	12.2	14.9	13.7	10.1	7.7	8.6	9.9
Air temperature average (°C)	22.1	16.2	16.6	19.8	19.7	20.7	19.3	15.4	18.8	15.7	16.8	14.8	15.7	16.1
Air temperature max (°C)	30.3	23.4	18.4	26.7	26.4	28.1	26.6	14.9	28.7	18.3	24.6	22.4	24.8	25.0
Air humidity average (%)	65.0	80.0	92.0	77.0	74.0	74.0	81.0	81.0	66.0	87.0	71.0	70.0	69.0	67.0
Wind speed average (m/s)	0.8	1.0	0.3	0.7	0.9	0.8	0.6	0.7	0.9	0.3	0.6	1.1	0.8	0.9

**Umbria**	**U1**	**U2**	**U3**	**U4**	**U5**	**U6**	**U7**	**U8**	**U9**	**U10**	**U11**	**U12**	**U13**	**U14**
Precipitation (mm)	6.0	0.0	0.0	0.0	0.0	10.4	0.4	0.2	0.6	22.4	0.0	0.0	0.0	0.0
Air temperature min (°C)	18.7	15.0	16.4	15.7	16.7	18.7	17.9	14.1	13.3	16.2	14.3	12.6	12.3	11.8
Air temperature average (°C)	23.6	20.5	20.8	22.8	23.3	24.2	23.0	18.1	20.4	21.0	20.2	19.2	19.0	19.1
Air temperature max (°C)	28.5	26.0	25.1	29.8	29.9	29.6	28.1	22.1	27.4	25.7	26.0	25.7	25.6	26.4
Air humidity average (%)	70.0	61.0	65.0	56.0	59.0	66.0	83.0	78.0	68.0	77.0	68.0	63.0	61.0	62.0
Wind speed average (m/s)	1.3	3.4	3.0	1.3	1.9	1.3	0.8	1.0	0.9	1.0	1.2	0.8	0.8	0.9

**Veneto**	**V1**	**V2**	**V3**	**V4**	**V5**	**V6**	**V7**	**V8**	**V9**	**V10**	**V11**	**V12**	**V13**	**V14**
Precipitation (mm)	0.0	0.0	0.0	0.0	0.0	0.0	0.0	0.2	0.0	0.0	0.0	0.0	0.0	0.0
Air temperature min (°C)	16.9	13.5	12.1	14.7	15.3	16.0	16.1	13.8	11.5	15.0	10.8	8.6	10.2	9.9
Air temperature average (°C)	22.3	20.0	21.1	22.2	23.2	23.5	23.2	19.3	18.2	16.8	16.5	16.2	16.9	16.8
Air temperature max (°C)	27.9	28.6	29.1	29.9	31.5	31.6	31.5	26.9	24.2	18.9	22.8	24.2	25.3	25.1
Air humidity average (%)	69.5	66.0	65.5	65.0	62.5	61.0	64.5	67.5	72.5	73.5	66.5	66.5	69.0	70.5
Wind speed average (m/s)	0.8	1.0	0.8	0.8	0.7	0.8	0.7	0.6	0.9	0.8	0.5	0.7	0.5	0.4
