# Supplementary material for: DNA metabarcoding uncovers fungal diversity of mixed airborne samples in Italy
Source: PLoS One. 2018 Mar 20;13(3):e0194489. doi: 10.1371/journal.pone.0194489 (PMC5860773; doi:10.1371/journal.pone.0194489)
Supplement: S5 Table — (PDF) [file pone.0194489.s009.pdf]

S5 Table. Land use according to CLC12 in 50 km buffers around each of the four sampling sites.

	FVG	MARCHE	UMBRIA	VENETO
**Artificial surfaces**	8.87%	3.54%	2.70%	11.25%
**Agricultural areas**	59.31%	55.80%	51.28%	65.23%
**Forest and semi natural areas**	28.49%	38.60%	45.20%	22.80%
**Wetlands**	0.73%	/	0.15%	0.21%
**Water bodies**	2.60%	2.06%	0.67%	0.51%
